# Simultaneous Imaging of Ribonucleic Acid and Hydrogen Sulfide in Living Systems with Distinct Fluorescence Signals Using a Single Fluorescent Probe

**DOI:** 10.1002/advs.201700966

**Published:** 2018-05-01

**Authors:** Yong Liu, Jie Niu, Weishan Wang, Yanyan Ma, Weiying Lin

**Affiliations:** ^1^ Institute of Fluorescent Probes for Biological Imaging School of Materials Science and Engineering School of Chemistry and Chemical Engineering University of Jinan Shandong 250022 P. R. China

**Keywords:** dual signals, hydrogen sulfide, organs, ribonucleic acid, tumors

## Abstract

Ribonucleic acid (RNA) and hydrogen sulfide (H_2_S) are important genes and gaseous signal molecules in physiological environment. However, simultaneous investigation of distribution and interrelation of RNA and H_2_S in living systems is restricted by lack of functional molecular tools. To address this critical challenge, the development of **TP‐MIVC** is described as the first paradigm of the probes that can concurrently report ribonucleic acid and hydrogen sulfide with distinct fluorescence signals in the cancer cells, zebrafish, and living animals. The advantageous features of the probe include high stability, low background fluorescence, high sensitivity, and two‐photon imaging property. Significantly, regardless of normal mice or tumor mice, tumor tissues exhibit stronger fluorescence intensity than other organs. More interestingly, it is found that **TP‐MIVC** is capable of distinguishing normal mice and tumor mice by in vivo imaging. This study may open a new pathway for distinguishing malignant and benign tumor by fluorescence imaging of RNA.

## 1 Introduction

Nucleic acids and hydrogen sulfide (H_2_S) have formed a sophisticated and frontier research field due to their pathological and physiological functions.^1^ Ribonucleic acid (RNA) plays central roles in converting genetic information from genes into the amino acid sequences of proteins.^2^ As a crucial gaseous transmitter, H_2_S is produced by cystathionine β‐synthase (CBS) and cystathionine γ‐lyase (CSE).^3^ The human gene for CSE is attached to the chromosome 1 (1p31.1) and two possible splice variants of CSE RNA have been confirmed.^4^ Similarly, scientists have confirmed that the human CBS gene is located on chromosome 21 (21q22.3)^5^ and encodes several RNAs, although the function of these RNA isoforms in terms of H_2_S synthesis is not known.^6^ That is to say, H_2_S could be produced by enzyme CSE and CBS are closely related to RNA. Thus, to better understand the function of RNA, it is important to develop single molecular tool that can simultaneously image RNA and H_2_S in biological systems.

Fluorescent probes are essential molecular tools for bioimaging.^7^ Nucleic acids were discovered by Friedrich Miescher in 1869.^8^ However, the nucleic acids were first used as probe in 1984.^9^ More recently, the well‐designed synthetic fluorescent nucleic acids dyes, including commercially available nucleic acids dyes, have been employed in bioimaging fields.^10^ Over the past few years, a wide variety of fluorescent H_2_S probes have been constructed.^11^ However, there have been no reports on probes for simultaneous imaging RNA and H_2_S in living systems. To dissect the complicated roles of H_2_S and RNA, it is highly valuable to create a single fluorescent probe that can simultaneously report RNA and H_2_S with distinct fluorescence signals. To the best of our knowledge, simultaneously reporting RNA and H_2_S is still an unmet challenge. At present, people could usually use a combination of an RNA‐specific dye indicator and a conventional H_2_S fluorescent probe to achieve this challenge; however, spectral cross‐talks, the distinct localization, and metabolisms may lead to failure of imaging.^12^

**Figure 1:**
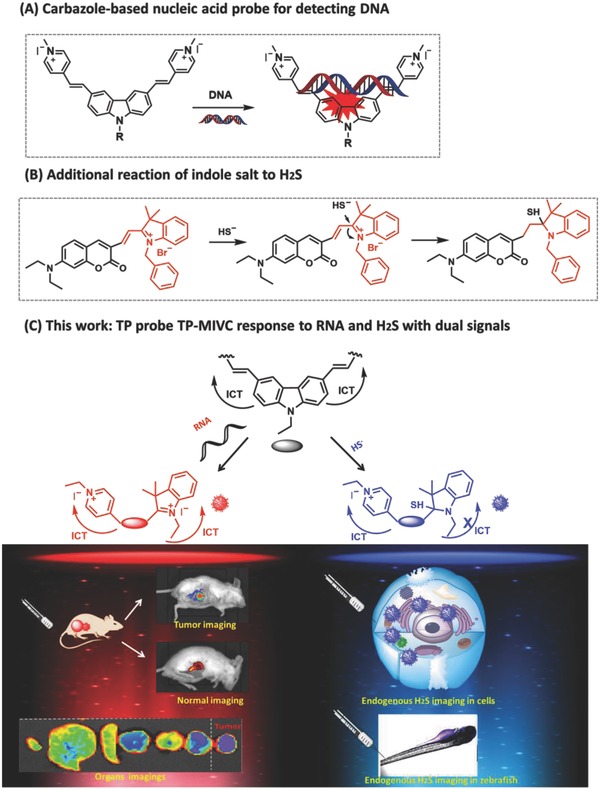
A) Carbazole‐based nucleic acid probe for detecting DNA. B) Sensing mechanism of indole salt to H_2_S. C) Rational design of the novel TP probe **TP‐MIVC** reporting RNA and H_2_S with two sets of different fluorescence signals.

Thus, our goal is to construct a single fluorescent probe, which could simultaneously image RNA and H_2_S with two different sets of fluorescence signals in living systems. The key of design to this unique probe should have two different sites that can react with RNA and H_2_S, respectively. Moreover, it should emit two distinct fluorescence signals in the presence of RNA and H_2_S, respectively. 3,6‐position substituted carbazole dyes could sense nucleic acids based on intercalative binding (**Figure** **1**A).^13^ Thus, we selected the carbazole 3‐(1‐methyl‐4‐vinylpyridiumiodine) carbazole (MVC) as fluorescent platform,^14^ in which a H_2_S site was introduced at the 3,6‐positions. In general, 3,6‐position substituted carbazole fluorescent probes display weak fluorescence signal in aqueous solution.^15^ In the presence of RNA, the intercalative binding of RNA to **TP‐MIVC** may induce a red fluorescence emission. Indolenium unit was selected as the site for H_2_S owing to its electrophilic character (Figure 1B).^16^ In the presence of H_2_S, the nucleophilic addition of HS^−^ to the indolenium C‐2 atom will provide **TP‐MIVC‐**SH^−^ with the overall conjugation breaking, which will decrease the intramolecular charge transfer (ICT) efficiency to induce a blueshift in the emission profiles. Thus, the probe **TP‐MIVC** would respond to RNA with a red fluorescence signal and H_2_S with a blue fluorescence readout.

In this study, the probe **TP‐MIVC** can simultaneously image RNA and H_2_S in cancer cells, zebrafish, and living animals with distinct fluorescence signals. Significantly, regardless of normal mice or tumor mice, tumor tissues exhibit stronger total, mean, and maximum and minimum fluorescence intensity than other organs. These results demonstrate that probe **TP‐MIVC** is capable of distinguishing normal mice and tumor mice (Figure 1C).

## 2 Results and Discussion

### 2.1 Photochemical Properties of the Free Probe TP‐MIVC

The synthetic route and the characterization data of the compound **TP‐MIVC** are shown in the Supporting Information (Scheme S1, Supporting Information). As shown in Figure S1 (Supporting Information), the free probe **TP‐MIVC** exhibited main absorption centered at 500 and 550 nm, and three relative weak absorption bands at 300–400 nm in various solutions. In phosphate buffer solution (PBS) buffer solution and water solution, the free probe **TP‐MIVC** shows low fluorescence quantum yield (*Φ* = 0.077 in water; *Φ* = 0.049 in PBS buffer solution) (Table S1, Supporting Information). In general, fluorescence dyes possessing A–π–D–π–A structures displayed weak fluorescence signal and low fluorescence quantum yield in aqueous solution due to twist ICT. We hope that the probe **TP‐MIVC** could display strong fluorescence signal and high fluorescence quantum yield in the presence of nucleic acid or active small molecules.

### 2.2 Response of Probe TP‐MIVC to RNA

**Figure 2:**
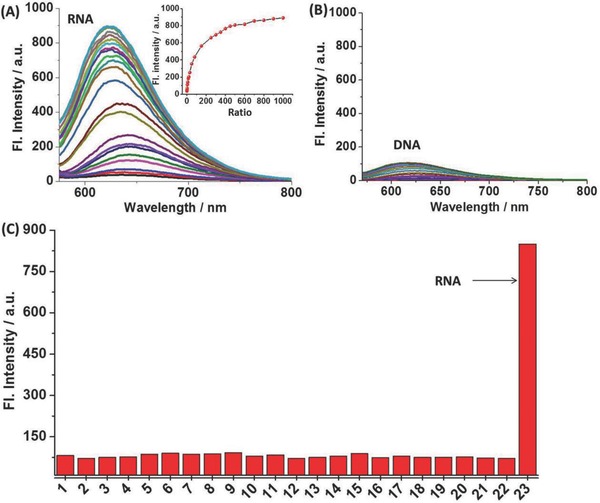
A) Fluorescence titration experiment of **TP‐MIVC** (10 × 10^−6^ m) in PBS buffer solution with the addition of RNA. Inset: Fluorescence variation trend with increasing of RNA. B) Fluorescence titration experiment of **TP‐MIVC** (10 × 10^−6^ m) in pure buffer solution with increasing DNA. C) Fluorescence responses of **TP‐MIVC** (10 × 10^−6^ m) to biological analytes in buffer solution. The concentrations of various biological analytes: amino acids, 1 × 10^−3^ m; GSH, 1 × 10^−3^ m; cations and anions, 2 × 10^−3^ m; and reactive oxygen and nitrogen species, 0.3 × 10^−3^ m. Legend: 1, **TP‐MIVC**; 2, Hcy; 3, GSH; 4, Ac^−^; 5, Br^−^; 6, Cl^−^; 7, CN^−^; 8, HPO_4_^−^; 9, HSO_3_^−^; 10; I^−^; 11, Mg^2+^; 12, PO_4_^3−^; 13, SO_3_^2−^; 14, SO_4_^2−^; 15, NO_2_^−^; 16, NO_3_^−^; 17, H_2_O_2_; 18, BSA; 19, anti‐EGFR; 20, glucose; 21, LPS; 22, tubulin; 23, RNA. λ_ex_: 488 nm.

To indicate **TP‐MIVC** can detect RNA, we set out to investigate the spectral changes of the probe in the presence of RNA in PBS buffer solution. The absorption and fluorescence spectra of **TP‐MIVC** in the absence and presence of RNA are shown in Figure S2 (Supporting Information) and **Figure** **2**A, respectively. Upon the addition of 1000 equiv. of RNA, the absorption band located at 481 nm did not increase obviously; however, the emission band centered at 625 nm increased gradually with the increasing of RNA at 488 nm excitation. Moreover, a 12‐fold enhancement in the fluorescence intensity was observed in the presence of RNA. The detection limit for RNA was obtained in buffer solution, and its detection limit is only 1.0 × 10^−6^ m (Figure S3, Supporting Information). The results demonstrate that the probe possesses high sensitivity in vitro. However, upon the addition of DNA, the fluorescence intensity of probe to DNA was not enough strong as probe to RNA (Figure 2B). Furthermore, similar to RNA, the emission band centered at 625 nm also presented upward tendency with the increasing of DNA under 488 nm excitation (Figure S4, Supporting Information). Fluorescence responses of **TP‐MIVC** to DNA and RNA indicated that the interference of DNA in vitro could be ignored (Figure S5, Supporting Information).

To evaluate the selectivity of the probe **TP‐MIVC** to RNA in PBS buffer solution, we selected a wide variety of possible competitive biologically relevant species, including Hcy, GSH, Ac^−^, Br^−^, Cl^−^, CN^−^, HPO_4_^−^, HSO_3_^−^, I^−^, Mg^2+^, PO_4_^3−^, SO_3_^2−^, SO_4_^2−^, NO_2_^−^, NO_3_^−^, H_2_O_2_, bovine serum albumin (BSA), anti‐ epidermal growth factor receptor (anti‐EGFR), glucose, lipopolysaccharide (LPS), and tubulin. As shown in Figure 2C, only RNA promoted obvious fluorescence intensity at 625 nm under 488 nm excitation, indicating the probe **TP‐MIVC** showed high selectivity for RNA in buffer solution.

### 2.3 Response of Probe TP‐MIVC to H_2_S

**Figure 3:**
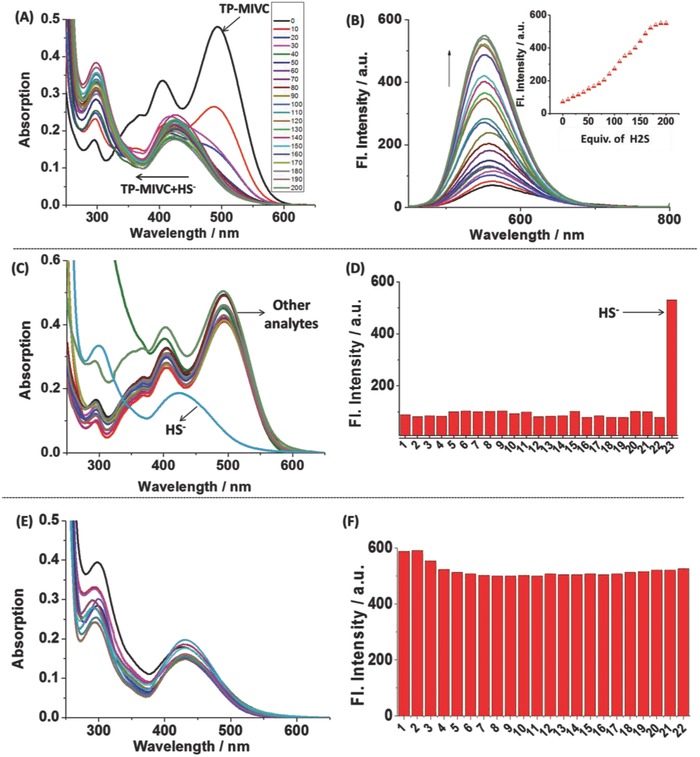
A) Ultraviolet titration experiment of **TP‐MIVC** (10 × 10^−6^ m) in pH 7.4 PBS buffer solution with the addition of H_2_S (0–200 equiv.). B) Fluorescence titration experiment of **TP‐MIVC** (10 × 10^−6^ m) in buffer solution with increasing of H_2_S (0–200 equiv.) at room temperature. Inset: Trend of fluorescence changes at 550 nm of **TP‐MIVC** (10 × 10^−6^ m) with increasing of H_2_S. C) Absorption spectra of **TP‐MIVC** (10 × 10^−6^ m) in the presence of various relevant analytes at room temperature. D) Fluorescence responses of **TP‐MIVC** (10 × 10^−6^ m) in the presence of various relevant analytes. Legend: 1, **TP‐MIVC**; 2, Br^−^; 3, Ca^2+^; 4, Cl^−^; 5, Cu^2+^; 6, Cys; 7, GSH; 8, H_2_O_2_; 9,Hcy; 10, HSO_3_^−^ 11, I^−^; 12, K^+^; 13, Mg^2+^; 14, Na^+^; 15, NO_2_^−^; 16, NO_3_^−^; 17, PO_4_^2−^; 18, S^2−^; 19, S_2_O_3_^2−^; 20, SO_3_^2−^; 21, SO_4_^2−^; 22, Zn^2+^; and 23, HS^−^. λ_ex_: 405 nm. E) Absorption spectra of **TP‐MIVC** (10 × 10^−6^ m) in pH 7.4 PBS buffer to HS^−^ in the presence of various analytes at room temperature. F) Fluorescence responses of **TP‐MIVC** (10 × 10^−6^ m) in pH 7.4 PBS buffer to HS^−^ in the presence of various analytes at room temperature. 1, **TP‐MIVC**‐HS**^−^**; 2, **TP‐MIVC**‐HS^−^ **‐**Br^−^; 3, **TP‐MIVC**‐HS^−^ **‐**Ca^2+^; 4, **TP‐MIVC**‐HS^−^ **‐**Cl^−^; 5, **TP‐MIVC**‐HS^−^ **‐**Cu^2+^; 6, **TP‐MIVC**‐HS^−^ **‐**Cys; 7, **TP‐MIVC**‐HS^−^ **‐**GSH; 8, **TP‐MIVC**‐HS^−^ **‐**H_2_O_2_; 9, **TP‐MIVC**‐HS^−^ **‐**Hcy; 10, **TP‐MIVC**‐HS^−^ **‐**HSO_3_^−^; 11, **TP‐MIVC**‐HS^−^ **‐**I^−^; 12, **TP‐MIVC**‐HS^−^ **‐**K^+^; 13, **TP‐MIVC**‐HS^−^ **‐**Mg^2+^; 14, **TP‐MIVC**‐HS^−^ **‐**Na^+^; 15, **TP‐MIVC**‐HS^−^ **‐**NO_2_^−^; 16, **TP‐MIVC**‐HS^−^ **‐**NO_3_^−^; 17, **TP‐MIVC**‐HS^−^ **‐**PO_4_^2−^; 18, **TP‐MIVC**‐HS^−^ **‐**S^2−^; 19, **TP‐MIVC**‐HS^−^ **‐**S_2_O_3_^2−^; 20, **TP‐MIVC**‐HS^−^ **‐**SO_3_^2−^; 21, **TP‐MIVC**‐HS^−^ **‐**SO_4_^2−^; 22, **TP‐MIVC**‐HS^−^ **‐**Zn^2+^.

To further investigate response of **TP‐MIVC** to H_2_S, the UV and fluorescence titration experiments were then carried out (**Figure** **3**A). The free probe **TP‐MIVC** itself exhibited a main absorption centered at 481 nm. Upon the addition of H_2_S, the absorption spectrum of probe generated blue‐shift. When the probe was excited at 405 nm, the fluorescence intensity evidently increased at 550 nm with the addition of HS^−^ (Figure 3B, inset). The detection limit for **TP‐MIVC** was calculated to be 3.2 × 10^−6^ m (Figure S6, Supporting Information), indicating that the probe is highly sensitive to H_2_S in PBS buffer solution. The formation of **TP‐MIVC**‐SH was confirmed using high resolution mass spectrum (HRMS) (Figure S7, Supporting Information). Moreover, the probe shows higher fluorescence quantum yield in the presence of H_2_S (*Φ* = 12.0) than in the absence of H_2_S (*Φ* = 0.049) (Table S2, Supporting Information). This result demonstrates the response of **TP‐MIVC** to H_2_S in PBS buffer solution. The absorption and fluorescence spectra of **TP‐MIVC** as a function of pH variations within 4.0–8.0 in an aqueous solution are shown in Figure S8 (Supporting Information). The emission band centered at 550 nm has no changes with the decreasing pH when the probe was excited at 405 nm. Thus, the results suggest that as anticipated the probe could report H_2_S in buffer solution with a blueshifted fluorescence signal, strong fluorescence readout at 550 nm.

Selectivity of the probe **TP‐MIVC** in pure water systems was further studied. Thus, we select a series of competitive relevant biological analytes to prove its selectivity in pH 7.4 PBS buffer solution. Biological thiol is a kind of biological molecule that may most likely interfere with the selectivity of the probe. Thus, we select the small‐molecule thiols such as cysteine (Cys), glutathione (GSH), and homocysteine (Hcy) to investigate the selectivity of the probe. In addition, other relevant species including the representative anions (Ac^−^, Br^−^, CN^−^, HPO_4_^−^, PO_4_^3−^, SO_3_^2−^, NO_3_^−^, Cl^−^, HSO_3_^−^, I^−^, NO_2_^−^, and SO_4_^2−^) and metal ions (K^+^, Mg^2+^, Ca^2+^, Na^+^, Cu^2+^, and Zn^2+^) are also selected. In the absorption spectrum, as shown in Figure 3C, the probe **TP‐MIVC** has an obvious blueshift in the presence of H_2_S. However, absorption spectrum of **TP‐MIVC** probe has no marked changes of absorption spectra in the presence of other relevant species. In fluorescence spectra, relevant species induced no marked fluorescence enhancement at relevant concentrations; however, an obvious enhancement in the fluorescence intensity was observed in the presence of H_2_S. The above results demonstrate the probe **TP‐MIVC** possesses higher selectivity to H_2_S over the analytes, which is further supported by the results of the competition experiments (Figure 3E,F). In addition, the probe exhibits high photostability in the presence of H_2_S in vitro (Figure S9, Supporting Information). Thus, we envision that this probe should show good photostability in the process of cells imaging.

We have proved that the probe **TP‐MIVC** could respond to RNA with a red fluorescence signal and an intense fluorescence readout at 625 nm. The probe **TP‐MIVC** could detect H_2_S using a blue fluorescence signal and a fluorescence readout at 550 nm. Thus, we believe that this single fluorescent probe, **TP‐MIVC**, can simultaneously image RNA and H_2_S in cancer cells with distinct fluorescence signals.

### 2.4 Simultaneous Imaging of RNA and Hydrogen Sulfide in Living Cells with Distinct Fluorescence Signals

**Figure 4:**
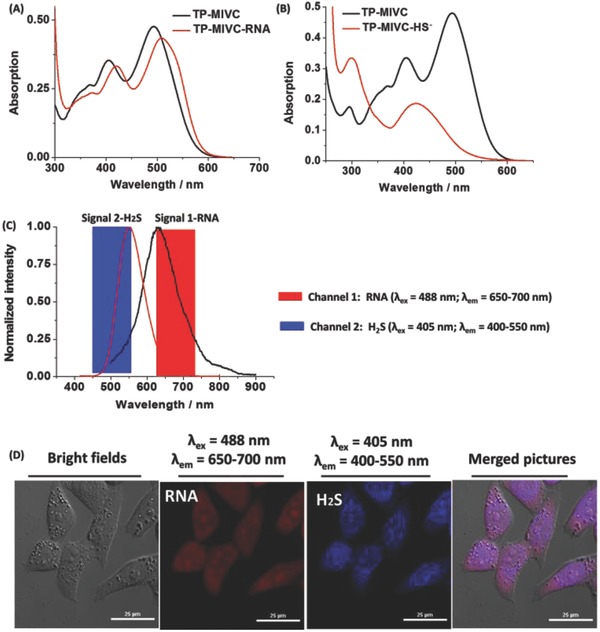
A) Absorption spectra of **TP‐MIVC** (10 × 10^−6^ m) in the absence and presence of HS^−^. B) Absorption spectra of **TP‐MIVC** (10 × 10^−6^ m) in the absence or presence of RNA. C) Fluorescence spectra of **TP‐MIVC** under 405 nm excitation in the presence of HS^−^, and fluorescence spectra of **TP‐MIVC** under 488 nm excitation in the presence of RNA. D) Confocal images of HeLa cells treated with **TP‐MIVC** (10 × 10^−6^ m) for 30 min. From left to right: Bright‐field image; the red emission channel (λ_ex_ = 488 nm, λ_em_ = 650–700 nm); the blue emission channel (λ_ex_ = 405 nm, λ_em_ = 400–550 nm); and merged image of (A) and (C). Scale bar = 25 µm.

In vitro, we have proved that probe **TP‐MIVC** could selectively sense RNA and H_2_S under different excitation wavelength. As shown in **Figure** **4**A,B, **TP‐MIVC** emitted similar absorption spectra in the absence and presence of RNA; **TP‐MIVC** showed different absorption spectra in the absence and presence of H_2_S. Interestingly, probe exhibited distinct fluorescence signals under 405 and 488 nm excitations (Figure 4C). Thus, we envision that probe **TP‐MIVC** is capable of imaging of RNA and H_2_S in living systems with distinct fluorescence signals.

**Figure 5:**
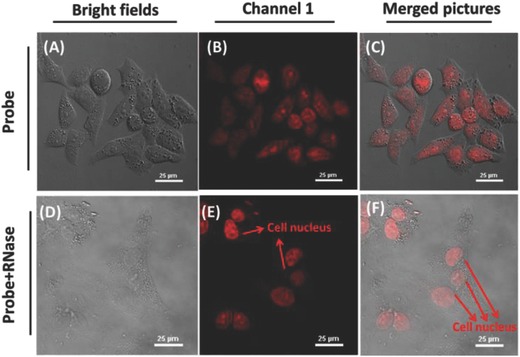
Fluorescence imaging of the endogenous RNA in HeLa cells using **TP‐ MIVC** in the absence or presence of RNase. A–C) The cells incubated with 5.0 × 10^−6^ m **TP‐MIVC** only. D–F) The cells pretreated RNase for 2 h and then incubated with 5.0 × 10^−6^ m **TP‐ MIVC**. A,D) Bright‐field images. C,F) Merged pictures. λ_ex_ = 488 nm; λ_em_ = 650–700 nm. Scale bar = 25 µm.

Encouraged by the above observations that the probe could report RNA and H_2_S in PBS buffer solution with intense fluorescence signals at 650 and 550 nm, respectively, we decided to examine whether the probe could sense RNA and H_2_S in living cells in dual‐color fluorescence imaging. To verify this conclusion, we set out to investigate the living cells imaging experiment. The cytotoxicity of the **TP‐MIVC** probe was evaluated using the standard 3‐(4,5‐dimethyl‐2‐thiazolyl)‐2,5‐diphenyl‐2‐*H*‐tetrazolium bromide (MTT) assays (Table S3, Supporting Information), indicating that the probe has low toxicity for the living cells. Thus, the living HeLa cells were incubated with the probe **TP‐MIVC**. As shown in Figure 4D, the cells exhibited strong fluorescence in the red (λ_ex_ = 488 nm, λ_em_ = 650–700 nm) and blue (λ_ex_ = 405 nm, λ_em_ = 400–550 nm) emission channels, in good agreement with the above findings in vitro. In the red channel, red fluorescence signals mainly focus on specific region, such as cytoplasm and nucleoli accompanied. As we all know, the nucleoli contains abundant proteins and RNAs, especially ribosomal proteins and ribosome RNA.^17^ Thus, red fluorescence signals of cells loaded with the probe may be caused by the endogenous RNA of cytoplasm and nucleoli. In the blue channel, blue fluorescence signals were located on cytoplasm. To prove the red fluorescence signal is indeed due to the imaging of the endogenous RNA in living cells, the digest test of ribonuclease (RNase) was carried out. Compared to HeLa cells untreated with RNase, living HeLa cells treated with RNase mainly localize at nucleoli (**Figure** **5**), in good agreement with commercially available RNA probe “SYTO RNA‐Select.”^18^ The results indicated that the probe was capable of imaging endogenous RNA in living cells.

Figure S10 (Supporting Information) exhibits the counterstain result of probe **TP‐MIVC** and DNA dye 4′,6‐diamidino‐2‐phenylindole (DAPI). The red fluorescence signals should be from nucleoli and cytoplasm of living cells. This signal is easily discriminated from the nuclear zone with blue fluorescence. This result demonstrates that **TP‐MIVC** has very good counterstain compatibility with DAPI. We deem that this probe may help image RNA distribution in relation to DNA in cells and probably to reveal different patterns of RNA‐DNA colocalization, which are cell type dependent. Furthermore, this probe exhibits high photostability in living cells (Figure S11, Supporting Information).

To further prove that fluorescence signal in the blue channel is from endogenous H_2_S of living cells, control experiments were then conducted. Phorbol myristate acetate (PMA) is known to reduce the endogenous H_2_S levels by promoting the generation of phagocytosis‐associated reactive oxygen species.^19^ Compared to cells untreated with PMA (Figure S12A,B, Supporting Information), the cells preincubated with PMA showed weak fluorescence in the blue channel (Figure S12C,D, Supporting Information). The mean fluorescence intensity with roughly tenfold weakens relative to the control group. Thus, the results of control experiments establish that the probe could image the endogenous H_2_S in living cells. We have proved that the probe possessed good stability in vitro. This probe still exhibits good photostability in living cells imaging. This result indicated that the probe is capable of real‐time imaging endogenous H_2_S in living cells (Figure S13, Supporting Information).

### 2.5 One and Two‐Photon Imaging Studies in Zebrafish with Distinct Fluorescence Signals

**Figure 6:**
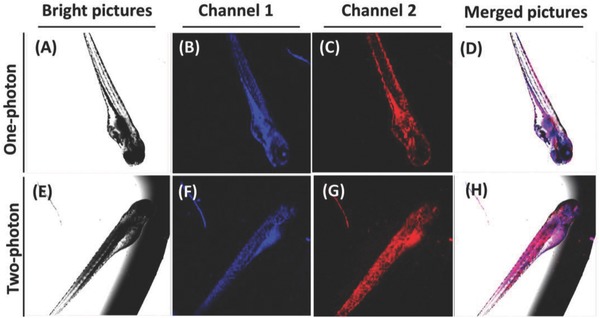
A–D) One‐photon fluorescence images and E–H) two‐photon fluorescence images of the zebrafish incubated with **TP‐MIVC** (5.0 × 10^−6^ m) for 1 h. A,E) Bright pictures; B,F) red emission channels: (one‐photon/two‐photon) λ_ex_ = 488/900 nm, λ_em_ = 650–700 nm. D,H) Merged pictures. C,G) Blue channel: λ_ex_ = 405/800 nm, λ_em_ = 425–475 nm.

The simultaneous one and two‐photon imaging of H_2_S and RNA in vivo was demonstrated using zebrafish as the vertebrate model. As shown in **Figure** **6**, **TP‐MIVC** loaded with zebrafish exhibited strong blue fluorescence and red fluorescence at one and two‐photon excitations. For one‐photon imaging, as shown in Figure 6A–D, both fluorescence signals were located on the chest of zebrafish. For two‐photon (TP) imaging, as shown in Figure 6E–H, the blue and red fluorescence signals mainly focus on the head, tail, and chest of zebrafish. The results demonstrated that the probe is suitable for TP imaging in zebrafish. We have demonstrated that the probe can simultaneously image RNA and H_2_S in living cells with distinct fluorescence signals. Thus, we envision that occurrence of both fluorescence signals was due to the response of probe to RNA and H_2_S. To the best of our knowledge, **TP‐MIVC** is the first paradigm of the probes that can concurrently report RNA and H_2_S with two different sets of fluorescence signals in zebrafish.

### 2.6 Distinguishing Normal Mice and Tumor Mice by In Vivo Imaging

**Figure 7:**
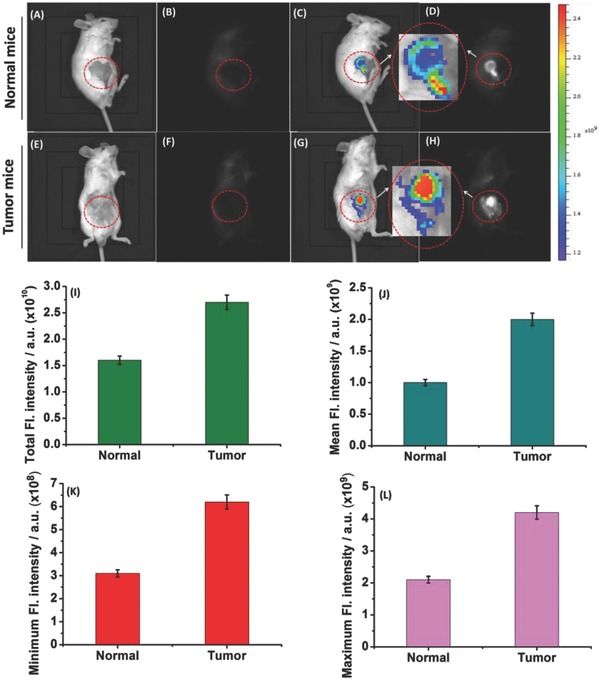
Control group (A)–(D): A) normal mouse only. B) Luminescence signal of normal mouse untreated **TP‐MIVC**. C) Fluorescence imaging of normal mice treated with **TP‐MIVC**. D) Luminescence of normal mouse treated with **TP‐MIVC**. Experiment group (E)–(H): E) tumor mouse only. F) Luminescence signal of tumor mouse untreated **TP‐MIVC**. G) Fluorescence imaging of tumor mouse treated with **TP‐MIVC**. H) Luminescence of tumor mouse treated with **TP‐MIVC**. I) Total and J) mean, K) minimum, and L) maximum fluorescence intensities in normal and tumor mice. In vivo fluorescence imaging of RNA in ((A) and (B)) normal mouse and ((C) and (D)) tumor mouse using **TP‐MIVC**. Control group ((A)–(D)) and experiment group ((E)–(H)): 80 µL of 500 × 10^−6^ m **TP‐MIVC** was injected for 30 min. λ_ex_ = 520 nm, λ_em_ = 620 nm. F) Minimum fluorescence intensities in various organs of normal and tumor mice. Error bars represent standard deviation (±S.D.).

Next, we further seek to assess in vivo images of **TP‐MIVC** in the living normal mice and tumor mice. As shown in **Figure** **7** (red region), normal mice and tumor mice were prepared. The normal mice untreated with **TP‐MIVC** could not emit strong fluorescence signal and luminescence signal (Figure 7A,B). The normal mice were injected with **TP‐MIVC** in an intraperitoneal (ip) manner. The normal mice treated with **TP‐MIVC** display essentially week fluorescence and luminescence signal (Figure 7C,D). Similar to normal mice, the tumor mice untreated with **TP‐MIVC** are almost impossible to generate fluorescence signal and luminescence signal (Figure 7E,F). However, the strong fluorescence and luminescence signals were obviously observed after the injection of **TP‐MIVC** within 30 min (Figure 7G,H). Interestingly, it is found that tumor mice treated with **TP‐MIVC** emit stronger fluorescence signals than normal mice (inset of Figure 7). To prove this point, total fluorescence intensity of normal and tumor mice has been obtained by us (Figure 7I). The result indicates that tumor mice treated with **TP‐MIVC** exhibit stronger fluorescence emission than normal mice. Furthermore, mean intensity and maximum and minimum fluorescene intensity of normal and tumor mice also further prove the above conclusion. Thus, the above results demonstrate that the probe **TP‐MIVC** is capable of distinguishing normal and tumor mice by in vivo fluorescence imaging (Figure 7J–L). We have proved that red fluorescence signal is from the response of probe to RNA in living cells. Thus, we envision that normal and tumor mice imaging may be from the response of probe to the endogenous RNA. In other words, we envision that probe **TP‐MIVC** is capable of distinguishing normal mice and tumor mice by in vivo fluorescence imaging of RNA in normal mice and tumor mice.

**Figure 8:**
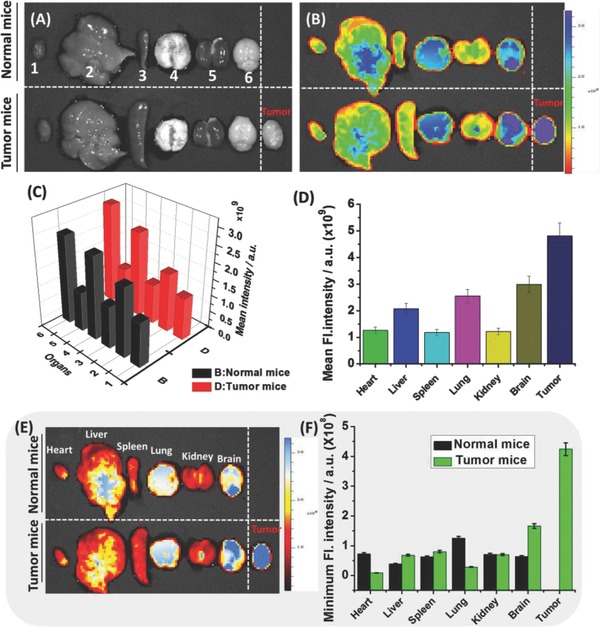
Fluorescence imaging of endogenous RNA with the probe **TP‐MIVC** in different mouse organs by an in vivo imaging system. A) Bright field images of mouse organs treated with 200 × 10^−6^ m probe, **1–6**: heart, liver, spleen, lung, kidney, brain, and tumor tissuess incubated with 200 × 10^−6^ m **TP‐MIVC**, respectively. B) Images of fluorescence and bright fields of mice organs treated with 200 × 10^−6^ m probe. λ_ex_ = 520 nm, λ_em_ = 620 nm. C) Mean fluorescence intensities in various organs of normal and tumor mice. D) Mean fluorescence intensities in various organs of tumor mice. Error bars represent standard deviation (±S.D.), *n* = 3. E) Images of fluorescence and bright fields of mice organs treated with 200 × 10^−6^ m probe. λ_ex_ = 520 nm, λ_em_ = 620 nm. F) Minimum fluorescence intensities in various organs of normal and tumor mice. Error bars represent standard deviation (±S.D.), *n* = 3. λ_ex_ = 520 nm, λ_em_ = 620 nm.

To further verify the above assumptiom, we decided to evaluate the ability of **TP‐MIVC** in various organs of normal and tumor mice. We required fluorescence imaging pictures of various organs (heart, liver, spleen, lung, kidney, brain, and tumor tissues) at 520 nm excitation (**Figure** **8**). As shown in Figure 8B,C, compared to the liver of tumor mice, normal mice exhibit stronger fluorescence signal and emission intensity. The brain of tumor mice shows higher fluorescence signal than normal mice. Fluorescence signals of the heart, spleen, lung, and kidney of normal mice are similar to tumor mice. Moreover, tumor tissues exhibit stronger mean fluorescence intensity than other organs (Figure 8D). To prove this point, minimum fluorescence emission of various organs of normal and tumor mice was further measured (Figure 8E,F). The results demonstrate that tumor tissues emit stronger minimum fluorescence signals than other organs. Furthermore, regardless of normal mice or tumor mice, tumor tissues exhibit stronger maxmium fluorescence intensity than other organs (Figures S14 and S15, Supporting Information). However, in normal and tumor mice, the liver shows stronger total fluorescence signal than other organs. The results demonstrate that probe **TP‐MIVC** is capable of distinguishing normal mice and tumor mice by in vivo fluorescence imaging of RNA. As we all konw, it is an important medical problem to distinguish malignant and benign tumor.^20^ Thus, this study may open a new pathway for distinguishing malignant and benign tumor by fluorescence imaging of RNA.

## 3 Conclusion

In summary, we have judiciously engineered a unique type of fluorescent probe represented by **TP‐MIVC**, which is capable of simultaneous imaging of RNA and H_2_S in different living systems with distinct fluorescence signals through rational design. This new probe shows excellent properties, including high stability, low background fluorescence, high sensitivity, and two‐photon imaging property. Furthermore, it is found that probe **TP‐MIVC** is capable of distinguishing normal mice and tumor mice by fluorescence imaging of RNA for the first time. This result may open a new pathway for distinguishing malignant and benign tumor. The dual‐signal fluorescence imaging studies of RNA and H_2_S illustrated herein may provide experimental and theoretical guide for further exploring the physiological functions of RNA and H_2_S in living animals.

## 4 Experimental Section

*Calculation of RNA/DNA Concentration*: RNA/DNA concentration in buffer solution was calculated by the Lambert–Beer law (1)

(1) $$A\;=\;-\log\frac{I}{I_{0}}\;=\;\varepsilon cl$$

where *A* is absorbance; ε is extinction coefficient; extinction coefficient of RNA is 7700 (DNA is 6600); *c* is molar concentration; *l* is the length of sample pool; and incident light and transmission light are *I*_0_ and *I*, respectively.

*Cell Culture and Live Cell Imaging*: **TP‐MIVC** was dissolved in dimethyl sulfoxide (DMSO) at a concentration of 1 × 10^−3^ m as stock solution. Cancer cells (HeLa) were cultured in Dulbecco's modified Eagle's medium supplemented with penicillin/streptomycin and 10% bovine calf serum in a 5% CO_2_ incubator at 37 °C. For duel‐channel imaging experiment of **TP‐MIVC**, cells were incubated with 5 × 10^−6^ m **TP‐MIVC** in culture medium for 30 min at 37 °C. After rinsing with PBS twice, HeLa cells were imaged immediately. Red channel: Fluorescence images of **TP‐MIVC** were collected between 650 and 700 nm under 488 nm excitation. Blue channel: Fluorescence images of **TP‐MIVC** were collected between 400 and 550 nm under 405 nm excitation.

*The PMA Control Experiment*: Two groups of living cells, control group and experiment group, were prepared. The two groups of cells were cultured in a 5% CO_2_ incubator at 37 °C for 2 d. First, for the experiment group, the culture medium of the cells was changed to a fresh medium containing 5 × 10^−6^ m **TP‐MIVC** and then incubated for 20 min. Subsequently, excess probes and medium were removed and washed three times with PBS buffer solution. Second, the culture medium of the one group cells was changed to a fresh medium containing 50.0 µL (3 µg mL^−1^) PMA and incubated for 3 h. Then, the medium was removed and this group of cells was washed three times with PBS buffer solution. After that, 1 mL of the medium containing 5 × 10^−6^ m **TP‐MIVC** was added and then incubated for 20 min. Finally, the confocal imaging was carried out using Nikon AMP1 two‐photon confocal fluorescence microscope.

*RNase Digest Test of Fixed Cells*: HeLa cells were grown on glass coverslips in a 5% CO_2_ incubator at 37 °C. After 2 d, two groups of HeLa cells were pretreated according to the following procedure: First, cells were fixed by 4% paraformaldehyde for 30 min and permeabilized by 0.5% Triton X‐100 for 2 min at room temperature. Second, one group of HeLa cells was incubated with 5 × 10^−6^ m **TP‐MIVC** for 30 min (as control group). Third, another group of cells was pretreated RNase (20 µg mL^−1^) for 2 h and then incubated with 5.0 × 10^−6^ m **TP‐ MIVC** (as experimental group). Finally, confocal fluorescence images were carried out using Nikon AMP1 confocal microscope with a 40× objective lens. Fluorescence images of **TP‐MIVC** were collected between 650 and 700 nm upon excitation at 488 nm.

*Fluorescence Imaging in Living Zebrafish*: First, zebrafish were purchased from Nanjing Eze‐Rinka Biotechnology Co., Ltd. Second, zebrafish were fed at 25 °C and a good breeding environment was created. Third, after 4 d, abundant zebrafish were obtained and further transferred into 30 mm glass culture dishes. Fourth, the probe **TP‐MIVC** (5 × 10^−6^ m) was added to the glass culture dishes containing zebrafish and incubated for 1 h. Fifth, after that, 1% agarose gel was adopted in small culture glass to fix living zebrafish. Finally, one‐photon and two‐photon fluorescence imaging were carried out. Dual‐channel fluorescence images were acquired using Nikon AMP1 confocal microscope with a 4× objective lens.

*Red Channel*: One‐photon fluorescence images of **TP‐MIVC** were collected between 650 and 700 nm upon excitation at 488 nm. Two‐photon fluorescence images of **TP‐MIVC** were collected between 650 and 700 nm upon excitation at 900 nm. Blue channel: One‐photon fluorescence images of **TP‐MIVC** were collected between 425 and 475 nm upon excitation at 405 nm. Two‐photon fluorescence images of **TP‐MIVC** were collected between 425 and 475 nm upon excitation at 800 nm.

*Preparation of Normal Mice/Organs and Tumor Mice/Organ Experiments*:^[21]^ The animal studies were permitted by the Institutional Animal Care and Use Committee of the Shandong University. All normal mice were obtained by School of Pharmaceutical Sciences, Shandong University. All normal mice were fed commercial mice chow and left freely wandering in their cage for 7 d for acclimatization before in vivo imaging experiment. All tumor mice were implanted 10^8^ number 4t‐1 cancer cells in subcutaneous tissue for 7 d.

For in vivo imaging, first, normal mice and tumor mice were given ip injection with 80 µL of **TP‐MIVC** (500 × 10^−6^ m). Second, normal and tumor mice were anesthetized with isoflurane. Third, to obtain accurate imaging results, abdominal fur must be shave cleaned using an electric shaver. Finally, in vivo images were carried out by an IVIS Lumina XR in vivo imaging system with an excitation filter of 520 nm and an emission filter of 620 nm.

For imaging various organs, first, normal mice and tumor mice were dissected and various organs were taken out, including the heart, liver, spleen, lung, kidney, brain and tumor tissues. Second, various organs were incubated with 200 × 10^−6^ m **TP‐MIVC** for 30 min. Finally, the imaging of organs was carried out by an IVIS Lumina XR in vivo imaging system with an excitation filter of 520 nm and an emission filter of 620 nm.

## Conflict of Interest

The authors declare no conflict of interest.

## Supporting information

Supplementary
