# Supplementary material for: Simultaneous Imaging of Ribonucleic Acid and Hydrogen Sulfide in Living Systems with Distinct Fluorescence Signals Using a Single Fluorescent Probe
Source: Adv Sci (Weinh). 2018 May 1;5(7):1700966. doi: 10.1002/advs.201700966 (PMC6051385; doi:10.1002/advs.201700966)
Supplement: Supplementary file 1 — Supplementary [file ADVS-5-1700966-s001.pdf]

Copyright WILEY-VCH Verlag GmbH & Co. KGaA, 69469 Weinheim, Germany, 2018.

ADVANCED SCIENCE Open Access

# Supporting Information

for *Adv. Sci.,* DOI: 10.1002/advs.201700966

# Simultaneous Imaging of Ribonucleic Acid and Hydrogen Sulfide in Living Systems with Distinct Fluorescence Signals Using a Single Fluorescent Probe

*Yong Liu, Jie Niu, Weishan Wang, Yanyan Ma, and Weiying Lin**

*Supporting Information*

# Simultaneous Imaging of Ribonucleic Acid and Hydrogen Sulfide in Living Systems with Distinct Fluorescence Signals Using a Single Fluorescent Probe

*Yong Liu, Jie Niu, Weishan Wang, Yanyan Ma, and Weiying Lin* *

Prof. Y. Liu, J. Niu, W. Wang, Y. Ma, Prof. W. Lin

Institute of Fluorescent Probes for Biological Imaging, School of Materials Science and Engineering, School of Chemistry and Chemical Engineering, University of Jinan, Shandong 250022, P.R. China.

E-mail: weiyinglin2013@163.com

# Table of content

1. Materials
2. Synthesis
3. Preparation of Samples and Test Solution (RNA)
4. Preparation of Samples and Test Solution ($H_2S$)
5. Quantum Yields
6. Cell viability Evaluated by MTT Assays
7. Figure S1.
8. Table S1.
9. Figure S2.
10. Figure S3.
11. Figure S4.
12. Table. S2
13. Figure S5.
14. Figure S6.
15. Table S3.
16. Figure S7.
17. Figure S8.
18. Figure S9.
19. Figure S10.
20. Figure S11.
21. Figure S12
22. Figure S13
23. Figure S14
24. Figure S15
25. Characterization

## 1. Materials

Firstly, for all experiment, all reagents of synthesis and analysis experiment were obtained by commercial suppliers. Before experiment, these reagents do not further purification. Furthermore, solvents were purified by standard methods before experiments.

Secondly, for synthesis experiment, all separation and purification of compounds were determined by TLC analysis. This method was performed on silica gel plates; In addition, column chromatography was carried out by silica gel (mesh 200–300); Silica gel was obtained from the Qingdao Ocean Chemicals.

Thirdly, for characterization of compounds, mass spectra were demonstrated by an LCQ Advantage ion trap mass spectrometer. It models is Thermo Finnigan or Agilent 1100 HPLC/MSD spectrometer; NMR spectra were obtained by the AVANCE III 400 MHz Digital NMR spectrometer.

Fourthly, for analysis experiment, ultraviolet absorption spectra were measured by a Labtech UV Power PC spectrometer; Fluorescence emission spectra were recorded with the HITACHI F4600 fluorescence spectrophotometer.

Fifthly, for biological imaging, fluorescence imaging of the cells and tissues slices was obtained with Nikon A1MP two-photon confocal microscopy. Two-photon imaging was conducted on with Nikon A1MP two-photon confocal microscopy (a Chameleon Vision II: Range 680~1080nm, a repetition rate of 80 MHz.). *In vivo* imaging was conducted on IVIS Lumina XR living animal imaging system.

## 2. Synthesis

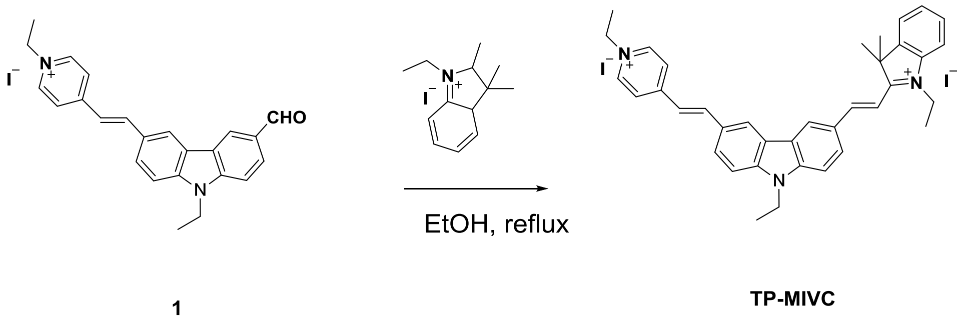


**Scheme. S1**. The synthetic route of the probe **TP-MIVC.**

*Synthesis routine of 1*: The compound 1 was synthesized by refer literature.[1]

*Synthesis routine of **TP-MIVC**:* Compounds 1 (0.540 g, 1.0 mmol) and iodoethane (0.190 g, 1.2 mmol) were dissolved in EtOH (15 mL); Mixture solution was allowed to react for 24 h at 80 $^{o}$C. EtOH in the mixture was evaporated, and then solid was washed by ether for several times. Finally solid was dried to afford the product **TP-MIVC** as a red solid with a yield of 85% $^{1}$H NMR (400 MHz, $CDCl_3$, $\delta$): $^{1}$H NMR (400 MHz, $CDCl_3$, $\delta$): 9.68 (s, 1H; Ar H), 9.09 (s, 1H; Ar H), 8.98 (s, 2H; Pyridine), 8.55 (d, *J* = 16.0 Hz, 1H; Ar H), 8.12 (d, *J* = 18.3 Hz, 2H; Ar H), 7.97 (s, 2H; Pyridine), 7.58 (d, *J* = 6.7 Hz, 5H; Ar H), 7.37 (d, *J* = 8.0 Hz, 2H; CH=CH), 7.24 (s, 1H; CH=CH), 7.15 (s, 1H; CH=CH), 5.08 (d, *J* = 8.1 Hz, 2H; $CH_2$), 4.77 (dd, *J* = 14.4, 7.5 Hz, 2H; $CH_2$), 4.31 (dd, *J* = 14.3, 7.4 Hz, 2H; $CH_2$), 1.95 (s, 6H; $CH_3$), 1.71 (d, *J* = 7.5 Hz, 6H; $CH_3$), 1.41 (t, *J* = 6.5 Hz, 3H; $CH_3$).$^{13}$C NMR (101 MHz, DMSO-*d6*, $\delta$): 181.24, 156.06, 153.61, 144.37, 144.11, 142.21, 141.03, 129.55, 128.48, 128.20, 128.00, 125.50, 123.66, 121.90, 121.16, 115.13, 111.47, 111.23, 109.61, 100.00, 60.83, 55.60, 52.40, 42.33, 38.37, 35.15, 26.46, 19.10, 16.69, 14.42, 14.25. HRMS (m/z): $[M\text{-}2I\text{-}H]^+/2$ calcd for $C_{37}H_{39}N_3^+$: 262.2000; found, 262.2817.

[1] F. Meng, Y. Liu, J. Niu, W. Lin, *RSC Adv.* **2017**, *7*, 16087.

## 3. Preparation of Samples and Test Solution (RNA)

(1) **TP-MIVC** was dissolved in DMSO to get 1 mM stock solution. 10 μM **TP-MIVC** were used in photophysical experiments. It was obtained by adding 50 μL the stock solution to 5.0 mL buffer solution.

(2) RNA/DNA solution: RNA/DNA concentration was calculated by the lambert beer's law (1).

$$A = -\log \frac{I}{I_0} = \varepsilon c l \quad (1)$$

(3) The solutions of various biological analytes were prepared from NaCl, KCl, $CaCl_2$, $MgCl_2$, glutathione (GSH), $H_2O_2$, homocysteine (Hcy), $Zn(Ac)_2$, KBr, KCN, $KHPO_4^-$,

$NaHSO_3$, $Ca_3(PO_4)_2$, KI, Cysteine (Cys), $NaNO_2$, $NaNO_3$, $Na_2S_2O_3$, $Na_2SO_3$, $Na_2SO_4$, BSA, Anti-EGFR, Glucose, LPS and Tubulin in PBS buffer solution. For the pH response experiments, the excitation wavelength was 488 nm, and the excitation and emission slit widths were 5 and 5 nm, respectively. Unless otherwise noted, for the titration and selectivity experiments, the excitation wavelength was 488 nm, and the excitation and emission slit widths were 5 and 5 nm, respectively.

**4. Preparation of Samples and Test Solution ($H_2S$)**

(1) **TP-MIVC** was dissolved in DMSO to get 1 mM stock solution. 10 μM **TP-MIVC** were used in photophysical experiments. It was obtained by adding 50 μL the stock solution to 5.0 mL buffer solution.

(2) The solutions of various biological analytes were prepared from NaCl, KCl, $CaCl_2$, $MgCl_2$, glutathione (GSH), $H_2O_2$, homocysteine (Hcy), $Zn(Ac)_2$, KBr, KCN, $KHPO_4^-$, $NaHSO_3$, $Ca_3(PO_4)_2$, KI, Cysteine (Cys), $NaNO_2$, $NaNO_3$, $Na_2S_2O_3$, $NaHSO_3$, $Na_2SO_4$, $Na_2SO_3$ and $Na_2S$ in PBS buffer solution. For the titration and selectivity experiments, the excitation wavelength was 405 nm, and the excitation and emission slit widths were 5 and 5 nm, respectively.

**5. Quantum Yields**

The fluorescence quantum yields are calculated by the following equation (1):

$$\Phi_s = \Phi_r \left( \frac{A_r(\lambda_r)}{A_s(\lambda_s)} \right) \left( \frac{n_s^{\ 2}}{n_r^{\ 2}} \right) \frac{F_s}{F_r} \qquad (1)$$

In the above equation, *s* and *r* stand for sample and the reference, respectively; *Φ* and *F* is quantum yield and integrated emission intensity, respectively. *A* and *n* is absorbance and refractive index, respectively.

6. **Cell viability Evaluated by MTT Assays**

Firstly, HeLa cells were fed in a 96-well plate in a 5% $CO_2$ incubator at 37°C.

Secondly, after 12 h, cells culture medium was changed to fresh medium containing different concenstration **TP-MIVC** (1, 5, 10, 20, 30, 50 μM).

Thirdly, after 24 h, the medium and the excess probe were removed, and then 10 μL 3-(4,5-

dimethyl-2-thiazolyl)-2,5-diphenyl-2-H-tetrazolium bromide (MTT) (5 mg/mL in PBS) was added to 96-well plate. Subsequently, the culture medium was removed, and 100 μL DMSO was added into the dishes to dissolve the formazan crystal product.

Fourthly, 96-well plate was shaken for a moment. Absorbance data at 490 nm was obtained by multiscan spectrum.

Finally, cell viability data was calculated by the following equation.

The cell viability (%) = ($OD_{490\ sample}$ - $OD_{490\ blank}$)/($OD_{490\ control}$ - $OD_{490\ blank}$) × 100%. $OD_{490\ sample}$ stand for the cells incubated with different concenstration probe, $OD_{490\ control}$ stand for cells untreated probe, $OD_{490\ blank}$ were wells containing culture medium only.

**7. Figure S1**.

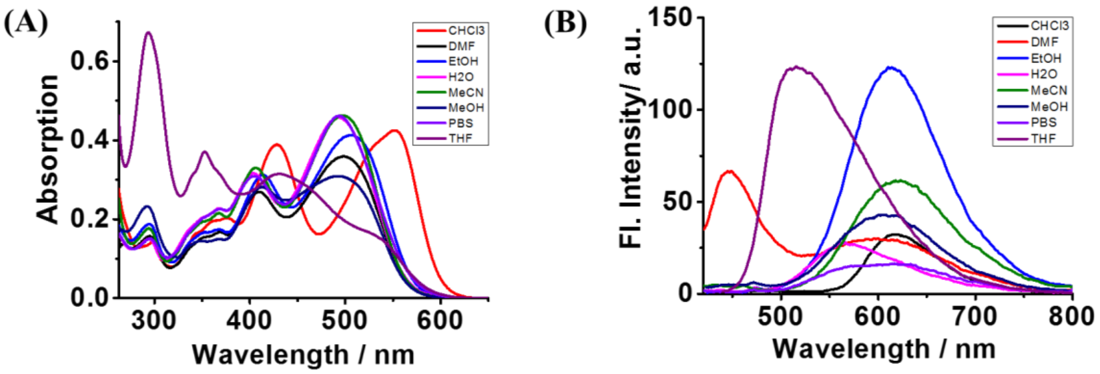


**Figure S1.** (A) Absorption spectra and (B) fluorescence responses of **TP-MIVC** in various solvents. [**TP-MIVC**]: 10 μM.

**8. Table S1.**

**Table S1.** The photophysical properties of **TP-MIVC** in various solvents.

| Solvents | $\lambda^a/\lambda^b$ (nm ) | $\varepsilon^c$ (×$10^4$) | $\Phi^d$ |
|---|---|---|---|
| PBS | 405/614 | 3.09 | 0.0493 |
| $H_2O$ | 405/566 | 3.16 | 0.0768 |
| $CHCl_3$ | 428/616 | 3.89 | 0.0657 |
| DMF | 410/600 | 2.69 | 0.0625 |
| MeOH | 413/604 | 2.82 | 0.1886 |
| MeCN | 405/618 | 3.30 | 0.2374 |
| EtOH | 414/610 | 3.12 | 0.4802 |
| THF | 430/516 | 3.15 | 0.5013 |

[a]Maximum absorption wavelength (nm). [b]Maximum fluorescence emission wavelength (nm). [c](×$10^4$ ) is molar absorptivity in $M^{-1}\cdot cm^{-1}$; [d]Φ is fluorescence quantum yield (error limit: 8%) determined by using fluorescein (*F* = 0.95) in NaOH aqueous solution (pH 11) as the standard.

**9. Figure S2**

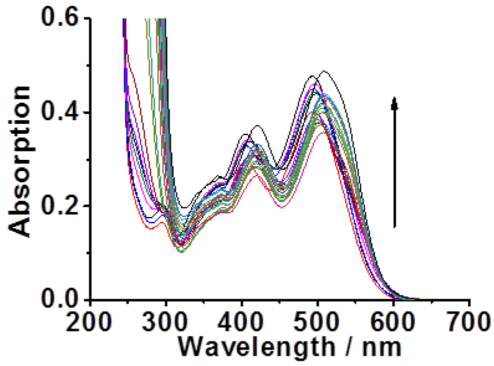


**Figure S2.** Ultraviolet titration expeiment of **TP-MIVC** with the increasing of RNA (0–100 equiv.) in PBS buffer solution. [**TP-MIVC**]: 10 μM.

**10. Figure S3.**

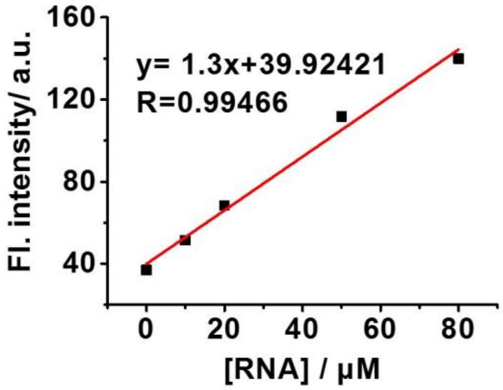


**Figure S3.** Normalized response of the fluorescence signal by changing the concentration of RNA.

**11. Figure S4.**

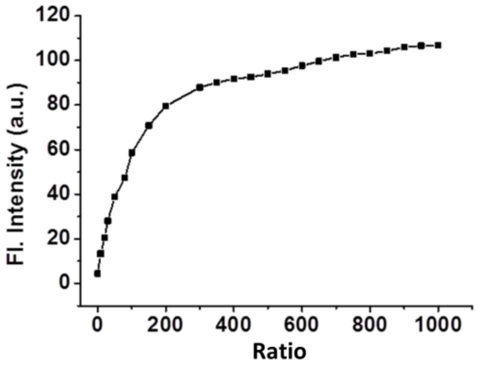


**Figure S4.** Fluorescence variation trend of **TP-MIVC** (10μM) with increasing of DNA. $\lambda_{ex}$: 488 nm.

**12. Figure S5.**

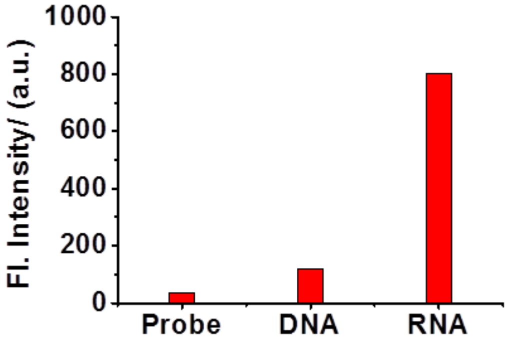


**Figure S5.** Fluorescence responses of **TP-MIVC** (10 μM) to DNA (400 equiv.) and RNA(400 equiv.) in buffer solution. $\lambda_{ex}$: 488 nm.

**13. Figure S6.**

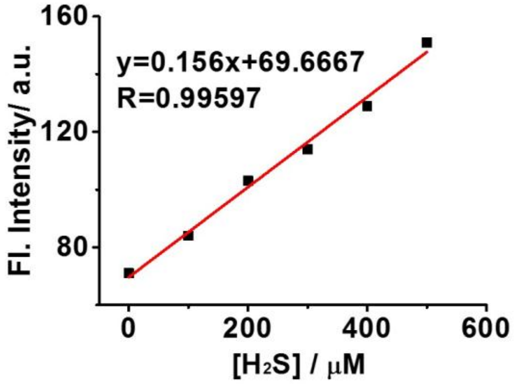


**Figure S6.** Normalized response of the fluorescence signal by changing the concentration of $H_2S$.

**14. Table. S2.**

**Table. S2** The photophysical properties of **TP-MIVC** in the presence and absence of $H_2S$

| | $\varepsilon^a$ ($\times 10^4$) | $\Phi^b$ (% ) |
|---|---|---|
| Probe | 3.09 | 0.049 |
| Probe+$H_2S$ | 1.79 | 12.0 |

[a]($\times 10^4$ ) is molar absorptivity in $M^{-1}\cdot cm^{-1}$; [b]$\Phi$ is fluorescence quantum yield (error limit: 8%) determined by using fluorescein (F = 0.95) in NaOH aqueous solution (pH 11) as the standard.

**15. Figure S7.**

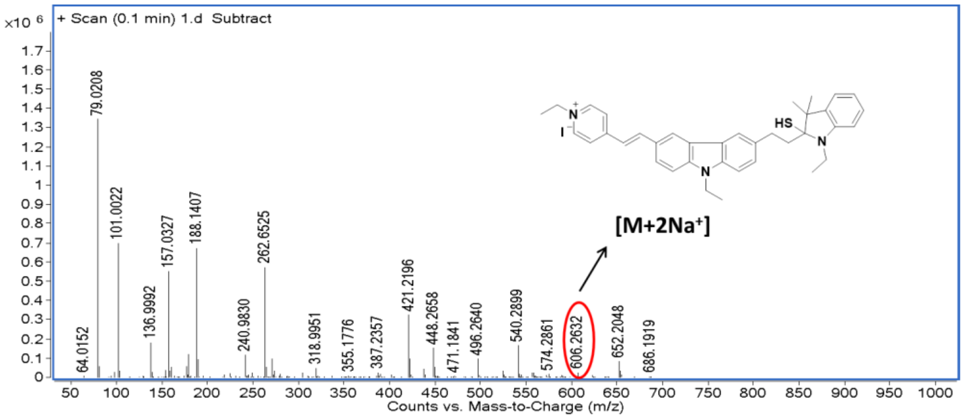


**Figure S7.** The formation of TP-MIVC -SH from the reaction of the probe with $H_2S$ was confirmed by HRMS. The intense peak at m/z 606.2632 in the HRMS spectrum corresponds to $(TP\text{-}MIVC\text{-}SH+2Na)^+$.

**16. Figure S8.**

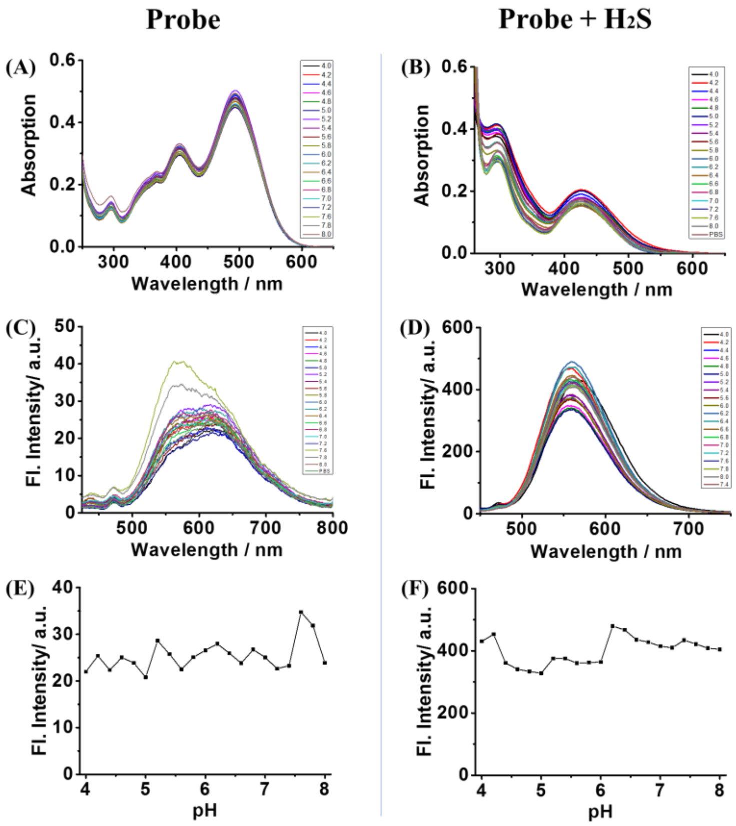


**Figure S8.** Absorption spectra of **TP-MIVC** (10 μM) in (A) the abscence and (B) presence $H_2S$ with different pH values (4.0-8.0); Fluorescence spectra of **TP-MIVC** (10 μM) in (C) the abscence and (D) presence $H_2S$ with different pH values (4.0-8.0). Changes of fluorescence spectra ($\lambda_{em}$ = 600 nm) of **TP-MIVC** (10 μM) in (E) the abscence and (F) presence $H_2S$ with different pH values (4.0-8.0).$\lambda_{ex}$: 405 nm.

**17. Figure S9.**

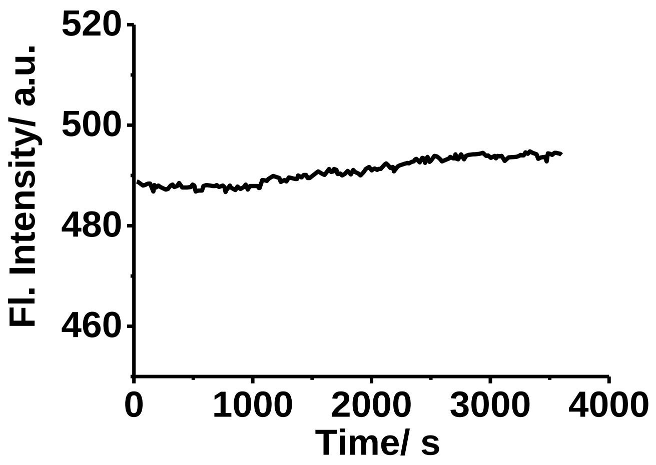


**Figure S9.** Fluorescence intensities of **TP-MIVC** to $HS^-$ in PBS buffer solution under successive irradiation for different times. conditions: [**TP-MIVC**] = 10 μM, $HS^-$: 200 equiv., $\lambda_{ex}$ = 488 nm.

**18. Table S3.**

**Table S3.** Cytotoxicity Data of **TP-MIVC (**10 μM) in HeLa cells[a].

| Incubate concentration(μM) | 1 | 5 | 10 | 20 | 30 | 50 |
|---|---|---|---|---|---|---|
| (% cell survival) | 100±4 | 98±4 | 96±4 | 90±4 | 84±4 | 78±4 |

[a] Cell viability was quantified by the MTT assays (mean ± SD).

**19. Figure S10.**

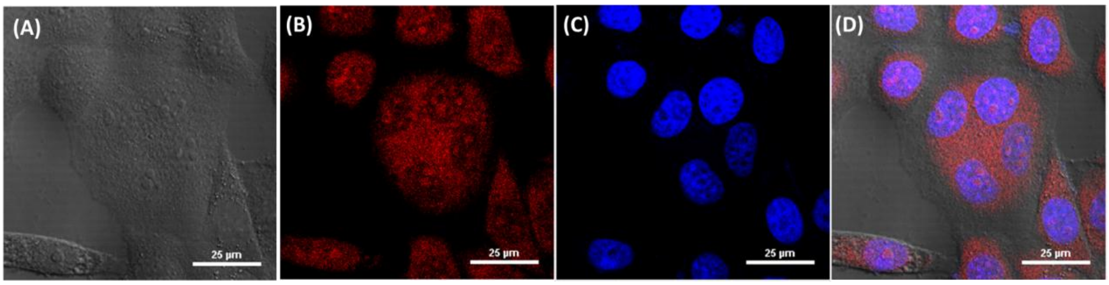


**Figure S10.** Fluorescence imaging pictures of HeLa cells incubated with 5 μM **TP-MIVC** for 30 min followed by incubating with 1 μM DAPI for 30 min. A: Bright field; B: Fluorescence imaging of **RNA**. C: Fluorescence imaging of DAPI. D: merged picture of A, B and C. Bar = 25 μm.

**20. Figure S11.**

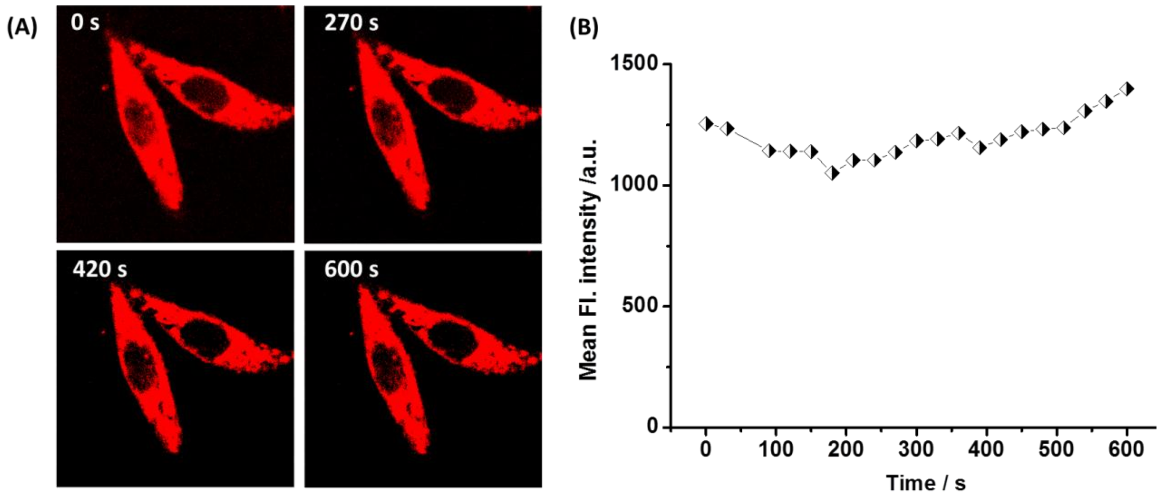


**Figure S11.** (A) Fluorescence images of HeLa cells incubated with **TP-MIVC** (5 μM) acquired at different times under successive excitation. (B) Mean intensities of the cells incubated with probe under successive excitation at different times. $\lambda_{ex}$: 488 nm, $\lambda_{em}$: 650-700 nm.

**21. Figure S12.**

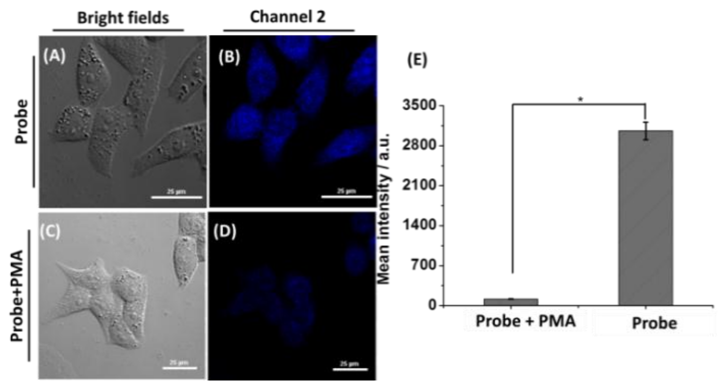


**Figure S12.** Fluorescence imaging of the endogenous $H_2S$ in HeLa cells using **TP-MIVC** in the absence or presence of $H_2S$ inhibitor. (A and B) the cells incubated with 5.0 μM **TP-MIVC** only; (C and D) the cells pretreated 50.0 μL (3 μg/mL) PMA for 3 h and then incubated with 5.0 μM **TP-MIVC**; (E) The comparison of the mean fluorescence intensities of B and D. Error bars represent standard deviation (±S.D.), n = 3

**22. Figure S13.**

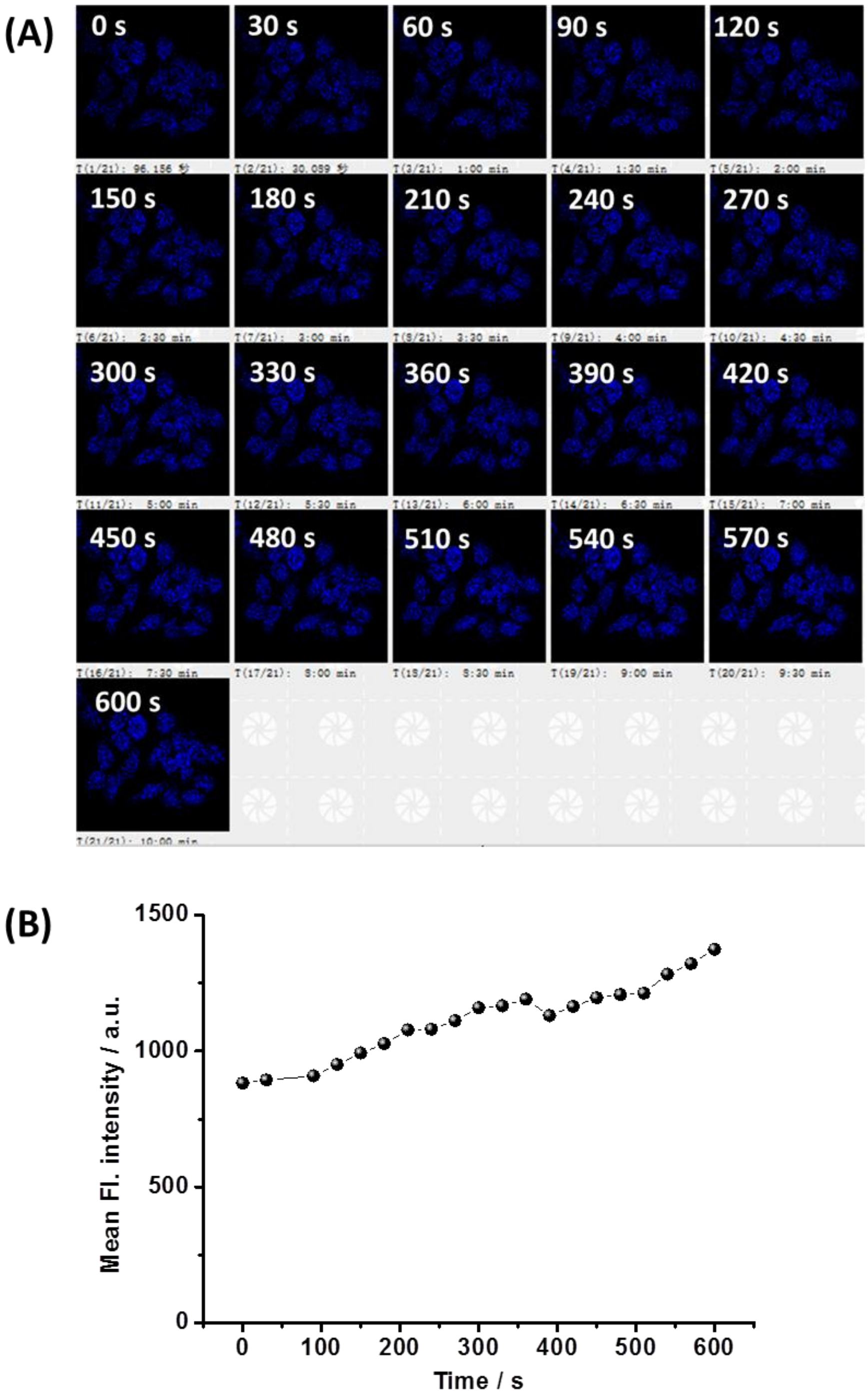


**Figure S13.** (A) Fluorescence images of HeLa cells incubated with **TP-MIVC** (5 μM) acquired at different times under successive excitation. (B) Mean intensities of the cells incubated with probe under successive excitation at different times. $\lambda_{ex}$: 405 nm, $\lambda_{em}$: 425-475 nm.

**23. Figure S14.**

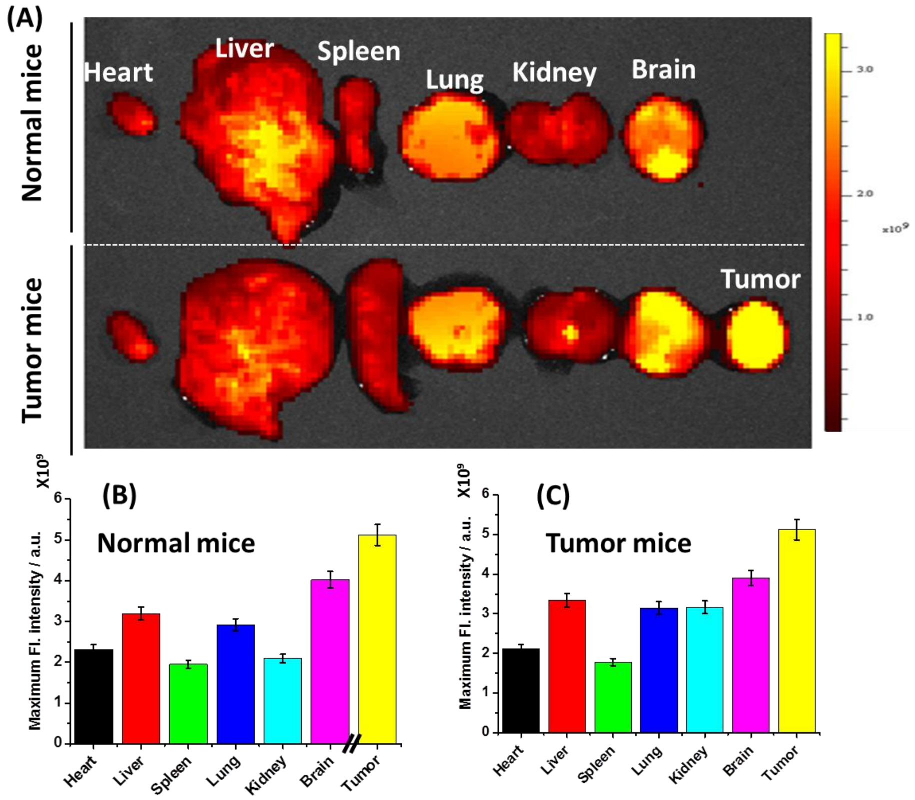


**Figure S14.** (A) Images of fluorescence of mouse organs treated with 200 μM probe. $\lambda_{ex}$ = 520 nm, $\lambda_{em}$ = 620 nm. (B) Maximum fluorescence intensities in various organs of normal mice. (C) Maximum fluorescence intensities in various organs of tumor mice. Error bars represent standard deviation (±S.D.), n = 3. $\lambda_{ex}$ = 520 nm, $\lambda_{em}$ = 620 nm.

**24. Figure S15.**

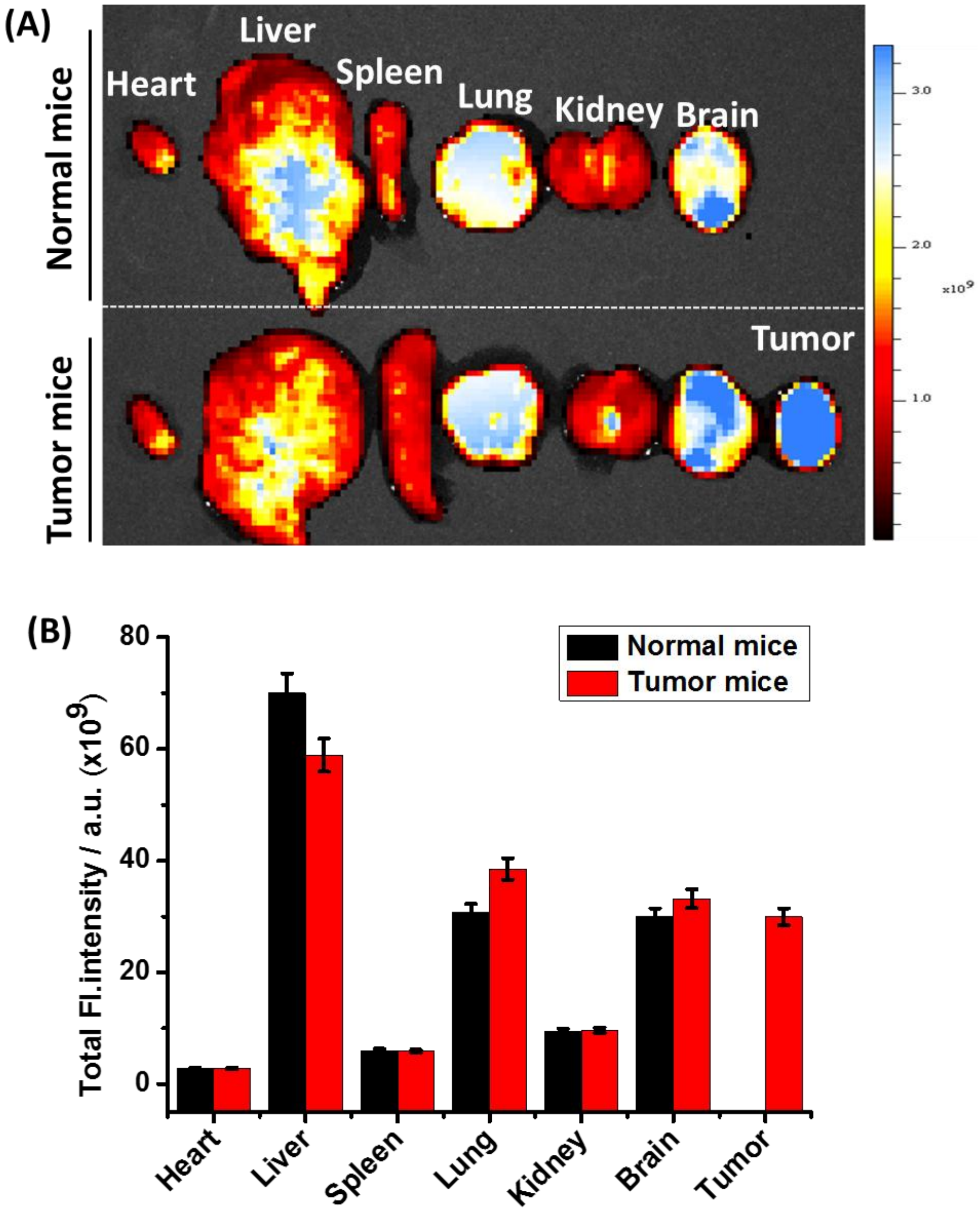


**Figure S15.** (A) Images of fluorescence of mouse organs treated with 200 μM probe. $\lambda_{ex}$ = 520 nm, $\lambda_{em}$ = 620 nm. (B) Total fluorescence intensities in various organs of normal and tumor mice. Error bars represent standard deviation (±S.D.), n = 3. $\lambda_{ex}$ = 520 nm, $\lambda_{em}$ = 620 nm.

**25. Characterization.**

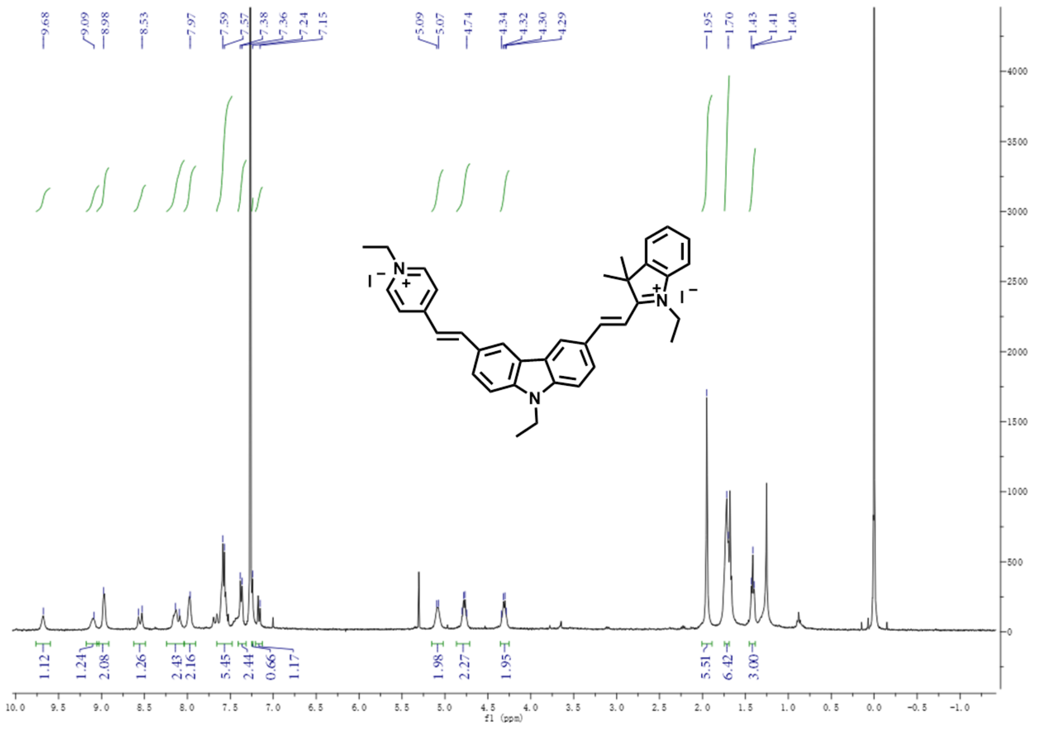

**Figure S16.** $^{1}$H NMR spectrum of **TP-MIVC**

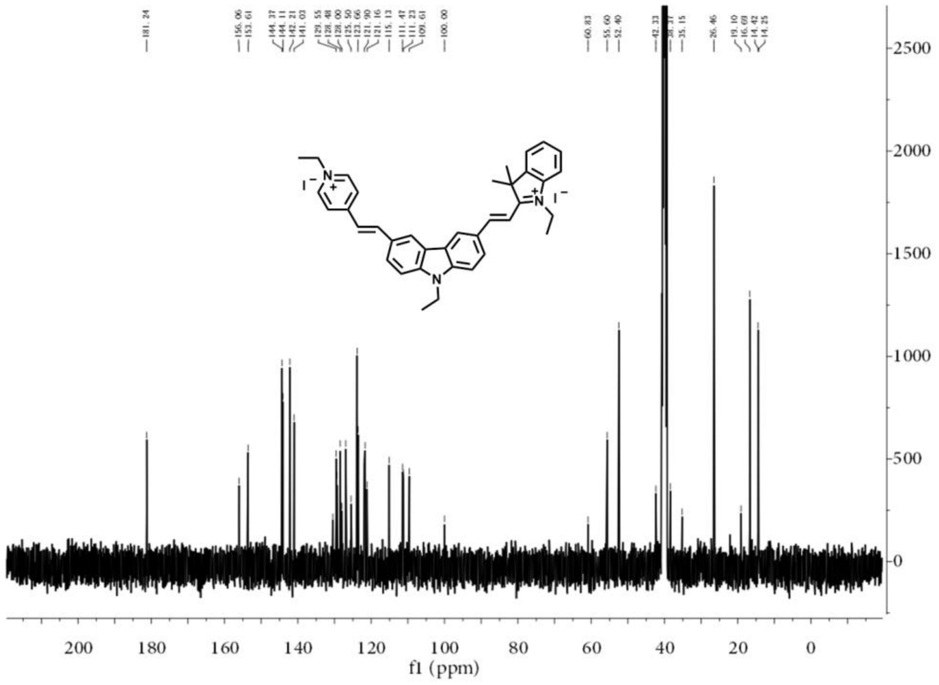

**Figure S17.** $^{13}$C NMR spectrum of **TP-MIVC**

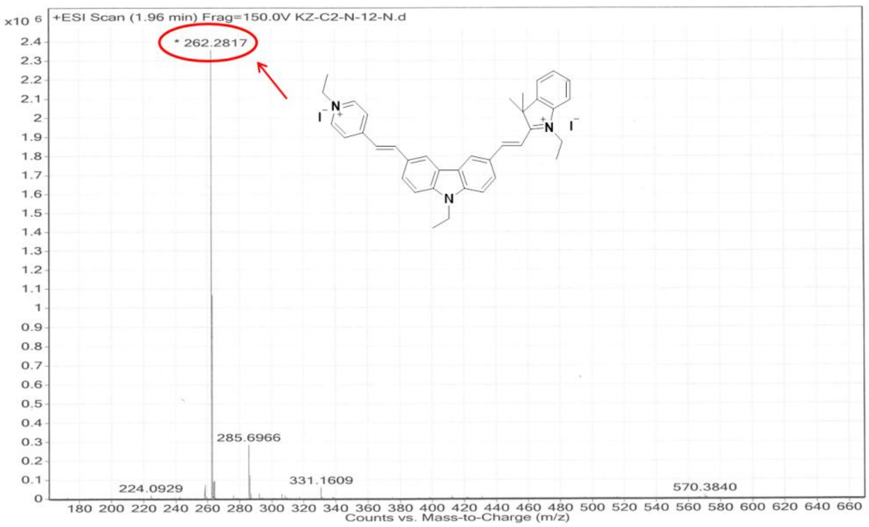


**Figure S18.** HRMS spectrum of **TP-MIVC**
